# A Technological Approach To Compensating for Cognitive Decline and Optimizing Healthcare Resources

**DOI:** 10.1002/alz70858_101225

**Published:** 2025-12-25

**Authors:** Valerie Larochelle, Quoc Dinh Nguyen, Julie Legault

**Affiliations:** ^1^ Eugeria, Montreal, QC, Canada; ^2^ Centre Hospitalier de l'Université de Montréal, Montreal, QC, Canada

## Abstract

**Background:**

The Idem Clock, a smart connected clock created by Eugeria Inc., enables caregivers to schedule reminders remotely and receive notifications if reminders are not confirmed. Through the MEDTEQ+ Innovative Health Showcase Program, the Montreal West Island Integrated University Health and Social Services Centre tested the clock to evaluate its value in home care, specifically its potential to reduce health and social service assistants’ visits for medication administration. The project also assessed secondary impacts on daily living activities and social isolation.

**Method:**

Over seven months, 48 older adults with mild to moderate cognitive impairments receiving home care services participated. Inclusion criteria included a Functional Autonomy Measurement System (FAMS) memory score of 0, ‐0.5, or ‐1, and a need for medication reminders. Exclusion criteria included severe memory impairment (FAMS score ‐2 or ‐3), Behavioral and Psychological Symptoms of Dementia (BPSD), or high‐risk medication use. Participants were evaluated by a nurse, who confirmed eligibility, obtained consent, and demonstrated the clock to users and caregivers. Installed in users’ homes, the clock displayed scheduled medication reminders. During the project, the nurse was responsible for scheduling medication reminders based on the participant's treatment. Nurses monitored compliance and received notifications when reminders were not confirmed. Performance was evaluated via feedback, re‐evaluations, and daily home visits.

**Results:**

Participants used the clock for an average of 66 days, with 81% continuing post‐trial. During the trial, 6,365 home visits were avoided, achieving a ratio of 53 visits avoided per programmed reminder. Each clock displayed an average of 68 reminders monthly (mean of 2 per day), with users confirming 80% of them. Estimated cost savings totaled CAD 80,842 for the healthcare center.

**Conclusion:**

The Idem Clock shows strong potential to enhance home care by reducing resource utilization and promoting self‐management for older adults with cognitive impairments. These findings support broader implementation, with recommended refinements to improve usability and application across diverse care settings.

